# Supplementary material for: PLK1 facilitates chromosome biorientation by suppressing centromere disintegration driven by BLM-mediated unwinding and spindle pulling
Source: Nat Commun. 2019 Jun 28;10:2861. doi: 10.1038/s41467-019-10938-y (PMC6599003; doi:10.1038/s41467-019-10938-y)
Supplement: Supplementary file 1 — Supplementary Information [file 41467_2019_10938_MOESM1_ESM.pdf]

Supplementary Information

PLK1 facilitates chromosome biorientation by suppressing centromere disintegration driven by BLM-mediated unwinding and spindle pulling

Addis Jones et al.

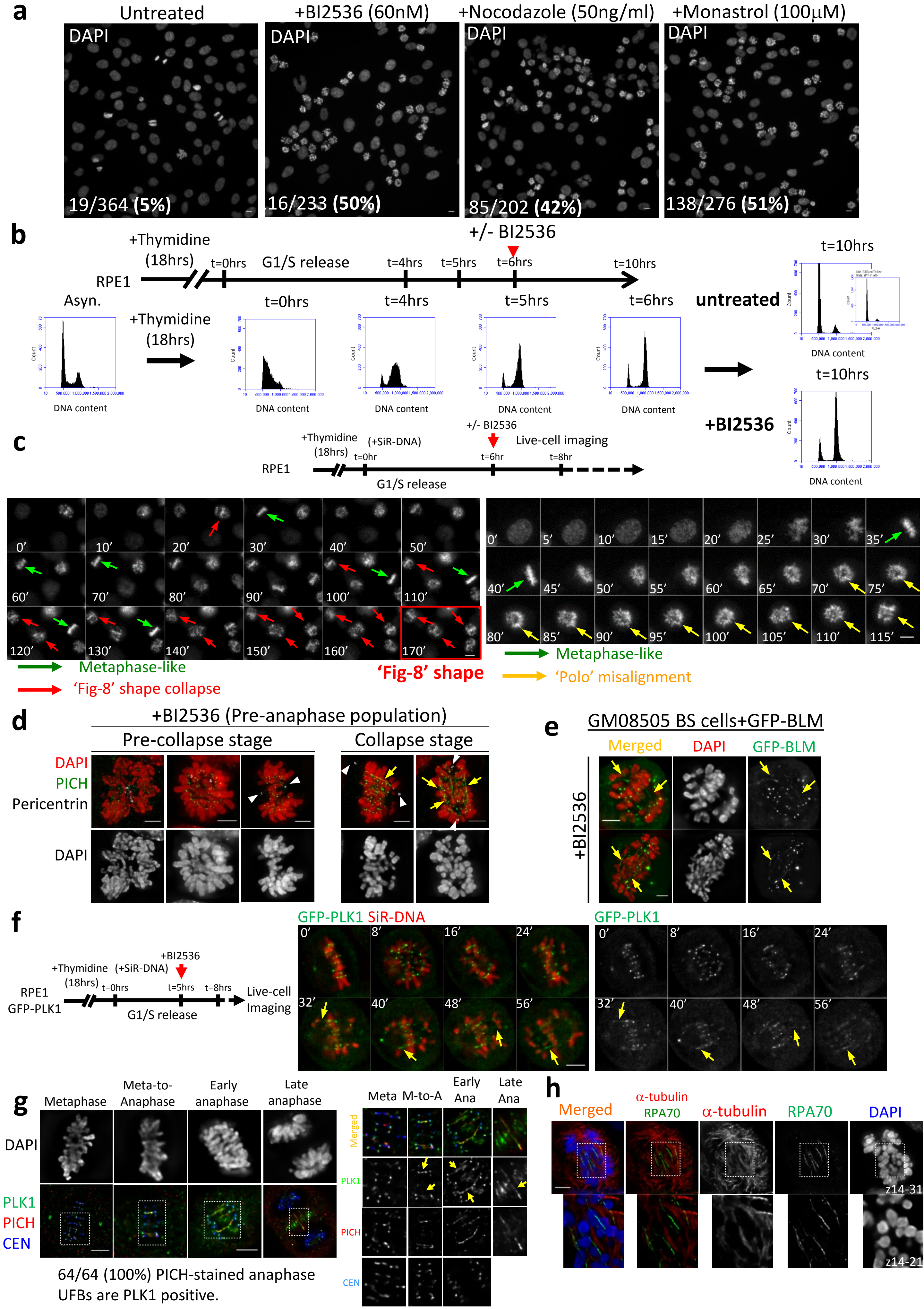

Supplementary Figure 1. Addis Jones *et al.*

Supplementary Figure 1. PLK1 inhibition using the small molecule inhibitor BI2536, induces metaphase collapse and the formation of centromeric DNA threads.

(a) Asynchronous RPE1 cells were treated with or without the indicated inhibitors for 18hrs and stained with DAPI. Indicated number of cells analysed and mitotic index are shown. Nuclei were identified by DAPI staining. Scale bar=10μm. **(b)** Experimental outline for FACS analysis of synchronised RPE1 cells with or without BI2536 treatment. Following 2mM thymidine treatment for 18hrs, samples were collected for FACS analysis at the indicated time points. BI2536 was added to a sample 6hrs post thymidine block release before FACS analysis. **(c)** Experimental outline and representative images of time-lapse live-cell microscopy of RPE1 cells released from single thymidine block, after BI2536 treatment. Left panels showing an example of three cells entering mitosis and progressing into a metaphase(-like) stage (green arrows), followed by metaphase collapse. Chromosome misalignment is reminiscent of a 'Figure-8' pattern (red arrows). Right panels showing a metaphase(-like) cell collapsing into a typical 'Polo' misalignment (yellow arrows). DNA was stained by SiR-DNA. **(d)** Representative images of RPE1 cells following cell synchronisation and treatment as in (b) showing both mitotic arrest of pre-collapse and collapse populations (yellow arrows denote PICH-coated thread-like structures; white arrowheads indicate centrosome positions). **(e)** EGFP-tagged BLM protein binds to DNA threads (arrows) in Bloom's syndrome fibroblasts (GM08505), following BI2536 treatment. **(f)** Experimental outline and time-lapse live-cell images on pre-synchronised RPE1 cells stably expressing GFP-tagged PLK1, highlighting metaphase collapse and pre-anaphase DNA thread formation (arrows), after BI2536 treatment (DNA was stained by SiR-DNA). **(g)** Pre-synchronised RPE1 cells showing mitotic progression from metaphase to anaphase and the presence of ultrafine-DNA bridge (UFBs) coated by PLK1 and PICH. Enlarged regions highlight UFB formation (arrows). 64/64, 100% of PICH-positive UFBs were positive for PLK1. **(h)** Representative image showing the distinct localisation pattern of DNA threads (RPA70), compared with microtubules (α-tubulin) in BI2536-treated RPE1 cells. Scale bars=5μm.

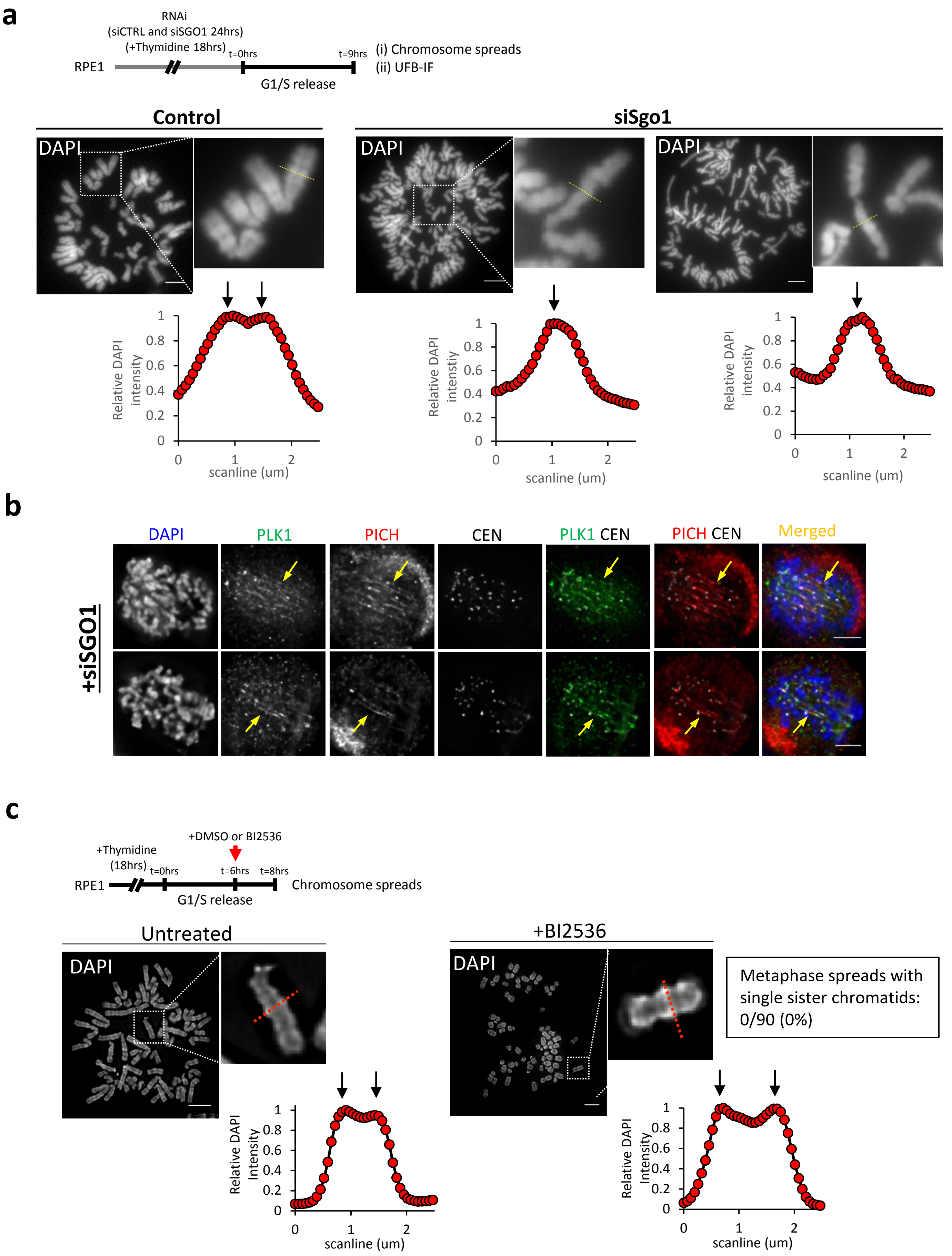

Supplementary Figure 2. Addis Jones *et al.*

Supplementary Figure 2. Shugosin 1 (SGO1) depletion, but not PLK1 inhibition, promotes premature sister-chromatid separation and the subsequent UFB formation.

(a) Experimental work flow of the cell synchronisation and SGO1 RNAi in RPE1 cells. Mitotic spreads were prepared and analysed for premature loss of sister chromatin cohesion. Representative images (top) and DAPI intensity plot profiles (below) indicated the precocious separation of sister-chromatids in Sgo1 depletion cells. **(b)** Representative images of immunofluorescent staining in SGO1-depleted cells showing the premature formation of PLK1- & PICH-associated UFB structures (yellow arrows). **(c)** Experimental work flow and representative images of mitotic spreads in untreated and BI2536 treated RPE1 cells. DAPI-intensity plot profiles indicate the existence of two peaks, highlighting that sister-chromatids remain cohesed in BI2536 treatment (0/90, 0% of spreads analysed showed signs of sister-chromatid separation). Scale bars=5μm.

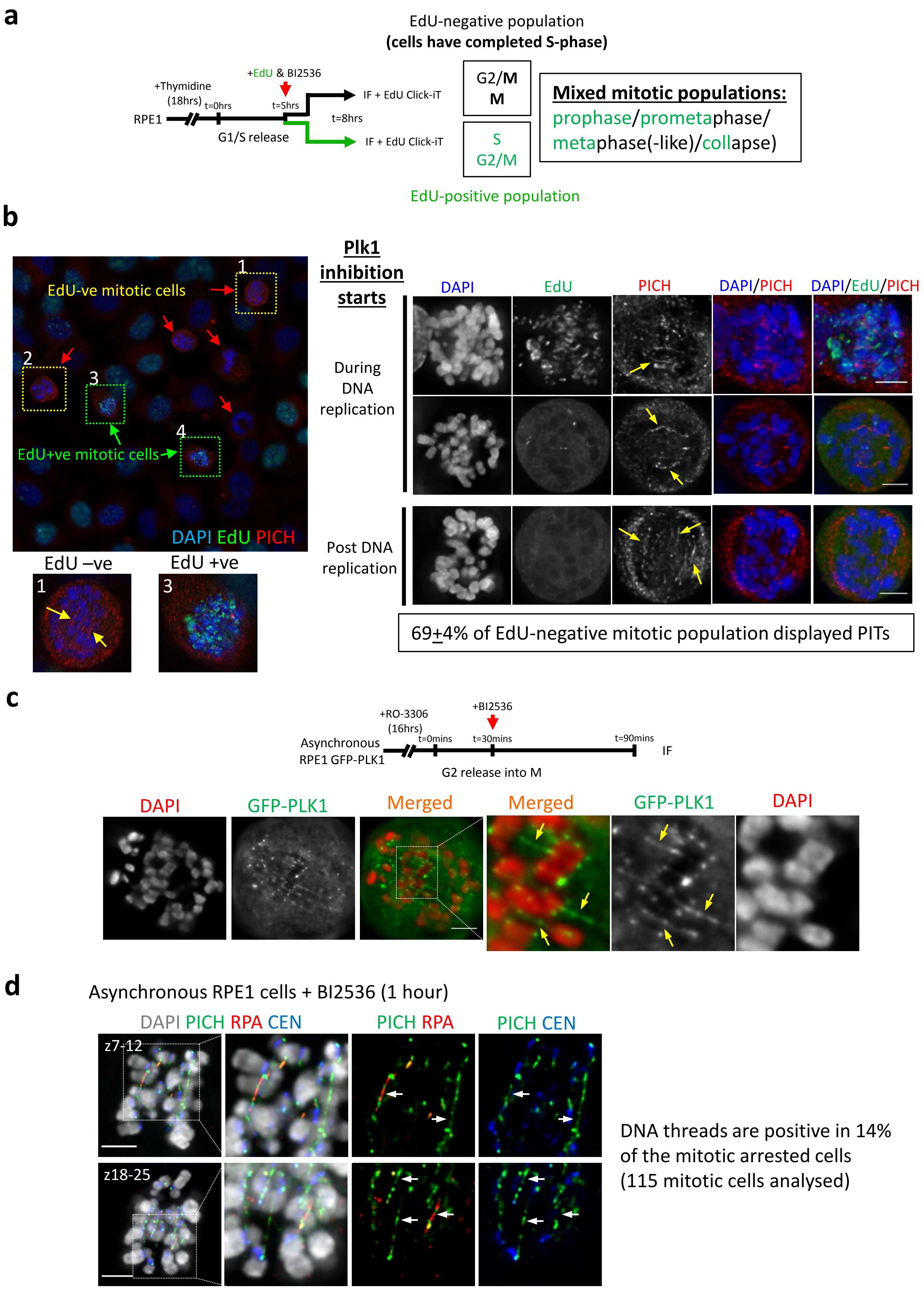

Supplementary Figure 3. Addis Jones *et al.*

Supplementary Figure 3. BI2536 induces centromeric DNA threads in post-DNA replication cells.

(a) Experimental workflow showing the labelling of S-phase cells with EdU while BI2536 treatment was carried out. It distinguishes the mitotic population that encounters PLK1 inactivation outside of S-phase. At 5hrs post single thymidine block release, RPE1 cells were treated with the thymidine analogue EdU, together with BI2536. After 3 hrs incubation, click-it chemistry and immunofluorescent staining were carried out. **(b)** Both EdU+ve and -ve mitotic populations exhibit DNA thread formation. An image (left) showing a mixture of mitotic cells that are either positive (green arrows; +ve), or negative (red arrows; -ve), for EdU signals. Representative images (right) of RPE1 cells displaying DNA thread formation (arrows) in both EdU-positive and negative cells. 69±4% of EdU negative cells are positive for DNA threads (mean± S.D. is shown). **(c)** Asynchronous GFP-tagged PLK1 RPE1 cells were arrested at G2 using the CDK1 inhibitor, RO-3306, for 16hrs. 30min post G2 release, BI2536 was added for 60mins followed by imaging. Representative image showing the formation of DNA threads bound by the GFP-PLK1 proteins. **(d)** BI2536 treatment in asynchronous RPE1 cells induced DNA thread formation. Asynchronous RPE1 cells were treated with BI2536 for 1 hour followed by immunofluorescent staining (115 mitotic cells were analysed). Scale bars=5μm.

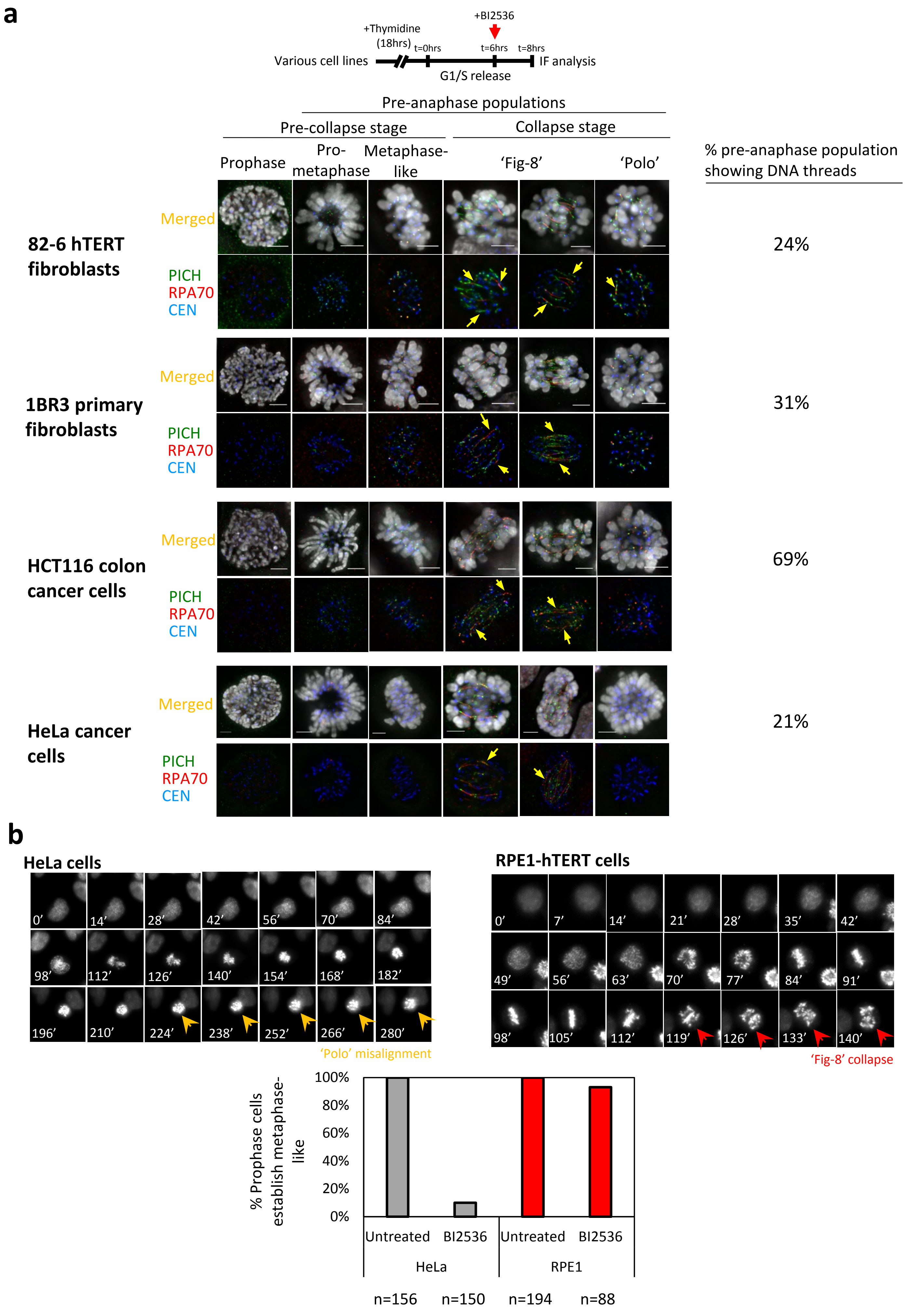

Supplementary Figure 4. Addis Jones *et al.*

Supplementary Figure 4. BI2536 induces centromere DNA thread formation in various human non-transformed and tumour-derived cell lines.

(a) Experimental workflow, representative images of different cell lines and their frequencies of DNA thread formation, after BI2536 treatment. Representative images of each cell line displaying the pre-anaphase mitotic populations from an early stage, denoted as pre-collapse (prophase to metaphase(-like), and collapse stages (both 'Fig-8' and 'polo') (70 of 82-6 cells, 80 of 1BR3 cells, 249 of HCT116 cells and 110 of HeLa cells were analysed). Scale bars=5μm. **(b)** Live-cell time-lapse microscopy showing the percentages of pre-synchronised RPE1 and HeLa cells establish metaphase(-like) stages during BI2536 treatment. Quantification is shown below. n=number of cells analysed. DNA was stained by SiR-DNA.

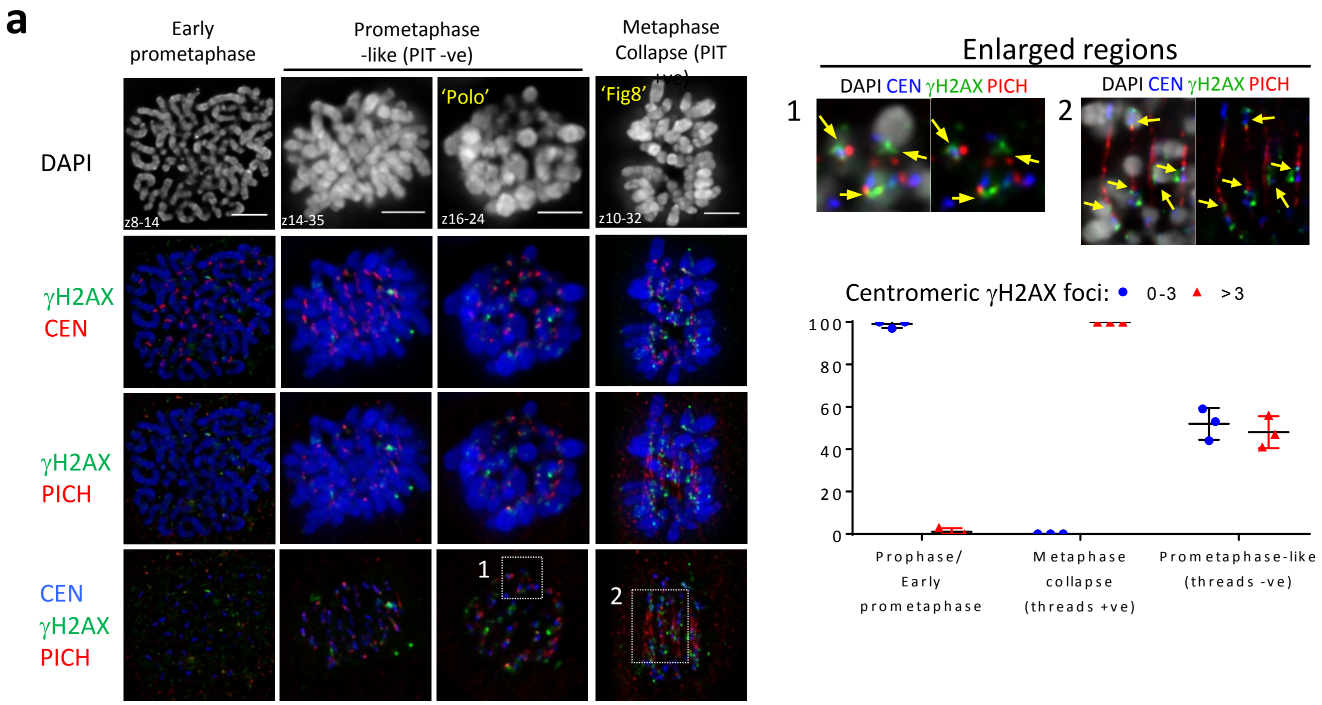

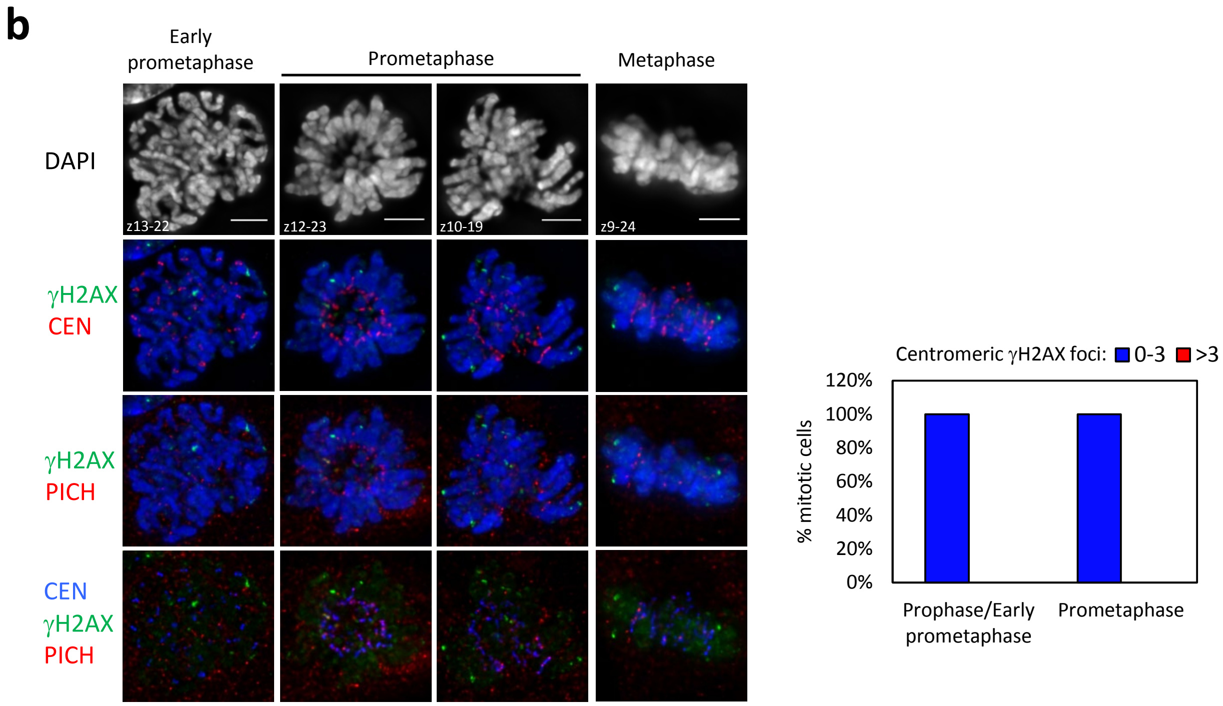

Supplementary Figure 5. Addis Jones *et al.*

Supplementary Figure 5. PLK1 inactivation triggers DNA damage response adjacent to centromeres.

(a) DNA damage responses occur at or surrounding centromeric regions following metaphase collapse and the formation of centromeric DNA threads. Representative images display different mitotic phases of RPE1 cells after BI2536 treatment and immuno-stained with PICH, γH2AX and CEN antibodies. Enlarged boxes denote γH2AX signals (arrows) at (peri)centromeric regions, which are either positive for PICH-coated DNA threads, or alternatively strong PICH foci. Quantitation of mitotic populations showing centromeric γH2AX foci (n=3 independent experiments analysing a total of 106, 103 and 96 cells in each stage of prophase/early prometaphase, metaphase collapse (thread +ve) and prometaphase-like (thread -ve); mean $\pm$ S.D. is shown). **(b)** Untreated mitotic populations display no sign of DNA damage response at centromeres, as demonstrated by an absence of γH2AX foci. Representative images of different mitotic phases of untreated (DMSO) RPE1 cells, immuno-stained with PICH, γH2AX and CEN antibodies. Quantification of mitotic populations showing centromeric γH2AX foci (n=23 and 21 cells analysed in prophase/early prometaphase and prometaphase populations, respectively, from one experiment). Scale bars=5μm.

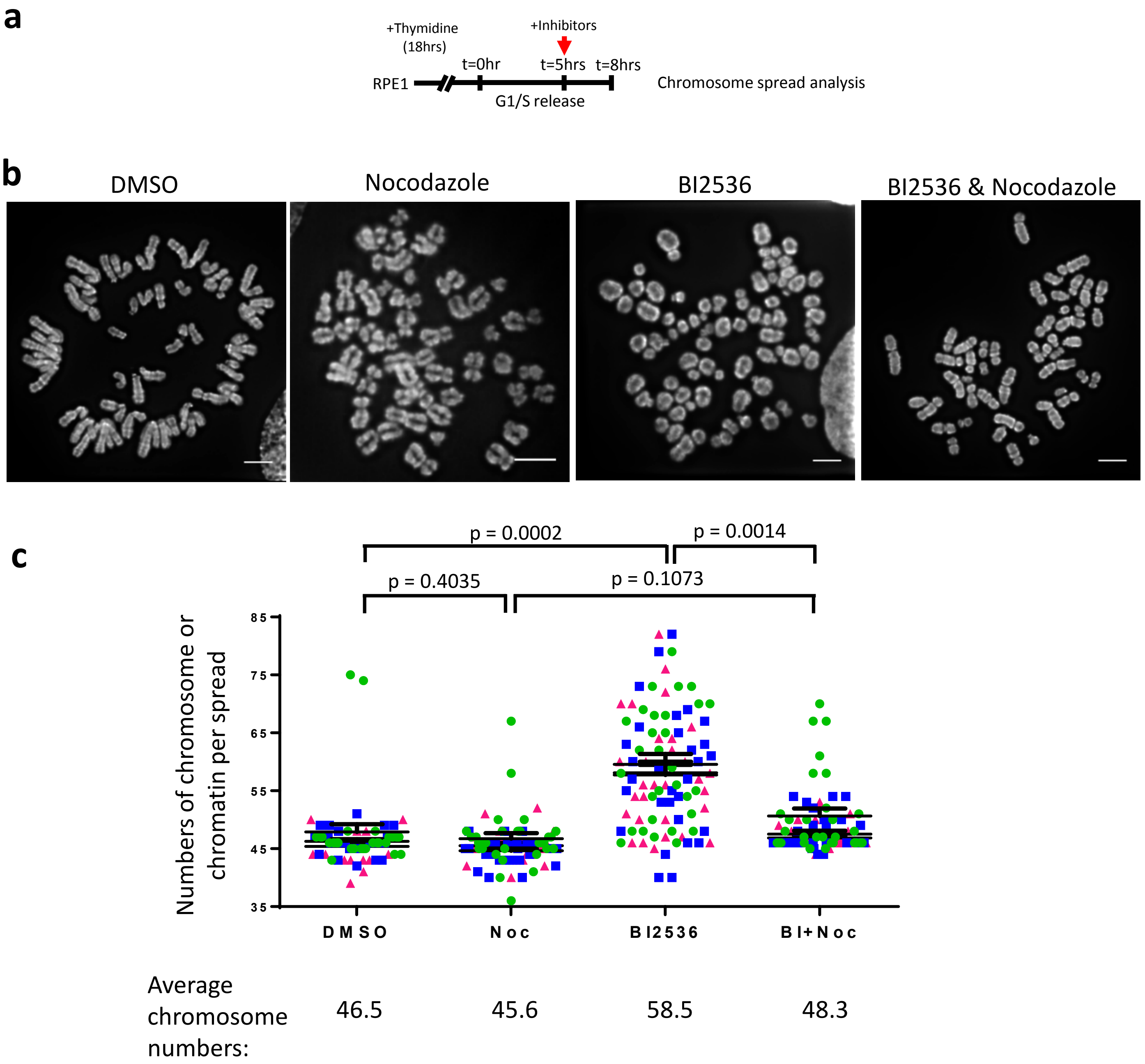

Supplementary Figure 6. PLK1 inactivation triggers mitotic chromosome fragmentation in a spindle-tension dependent manner.

(a) Experimental outline of cell synchronisation and treatment. **(b)** Representative images of mitotic chromosomes spreads after treatment with indicated inhibitors. Scale bar=5µm. **(c)** Quantification of chromosome/chromatin number of the indicated inhibitor treatment (average chromosome numbers is shown below; a total of 90 spreads were scored in each condition from three independent experiments).

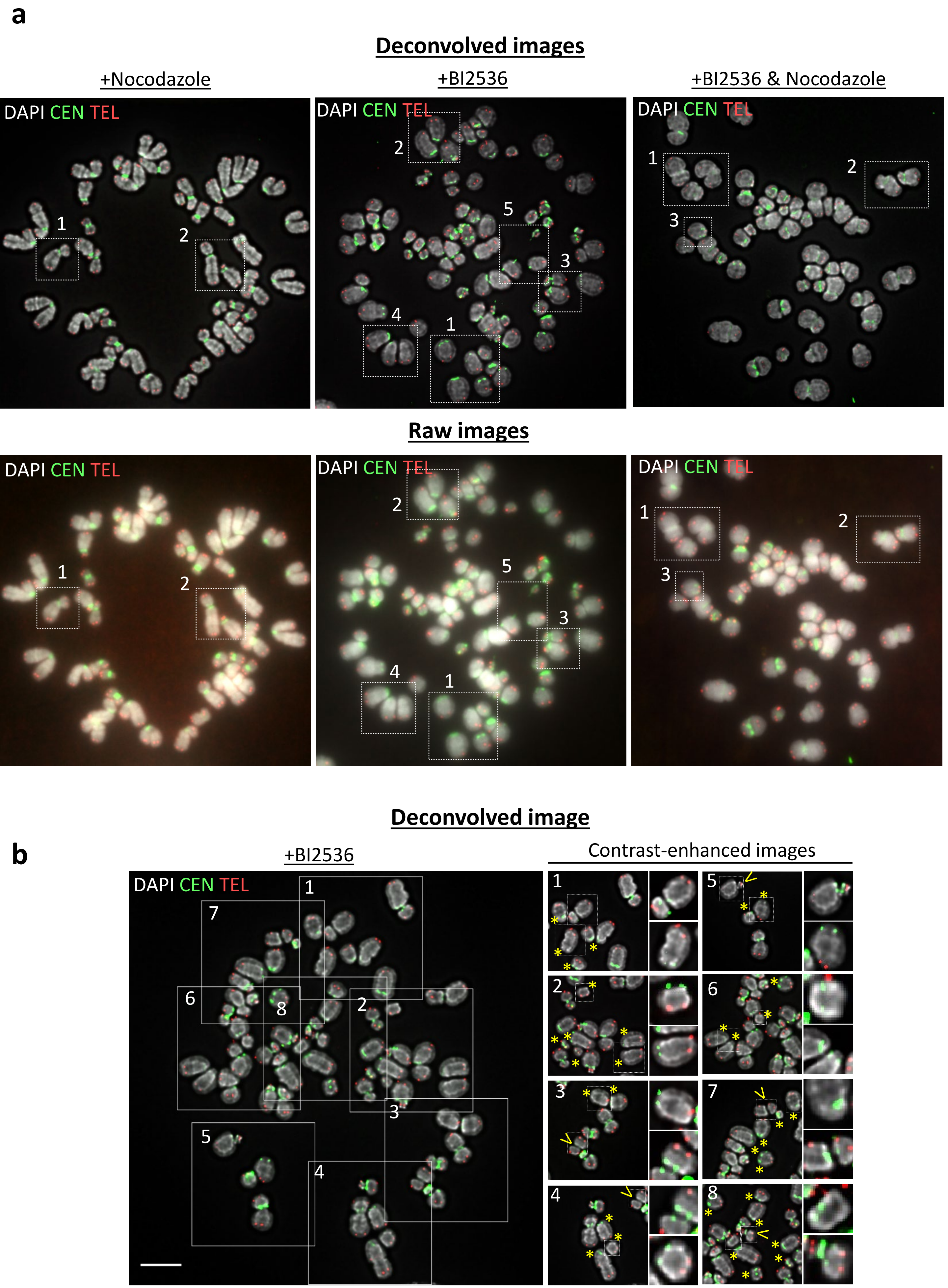

Supplementary Figure 7. Addis Jones *et al.*

Supplementary Figure 7. PLK1 inhibition specifically induces chromosome breakage at centromeres.

(a) Representative deconvolved and raw FISH images of chromosome spreads following treatment with indicated inhibitors (experimental set up as before from supplementary fig. 6a). Centromeres and telomeres were hybridised with FISH DNA probes. **(b)** Representative example of a BI2536-treated chromosome spread with chromosome breakage at centromeres. Enlarged (1-8; right) regions have been further cropped to allow for contrast enhancement of individual centromere FISH signal detection at each broken chromatin (note: in the absence of increased contrast, the centromere FISH signal can be difficult to see and therefore appear as missing (arrow heads). Scale bar=5μm.

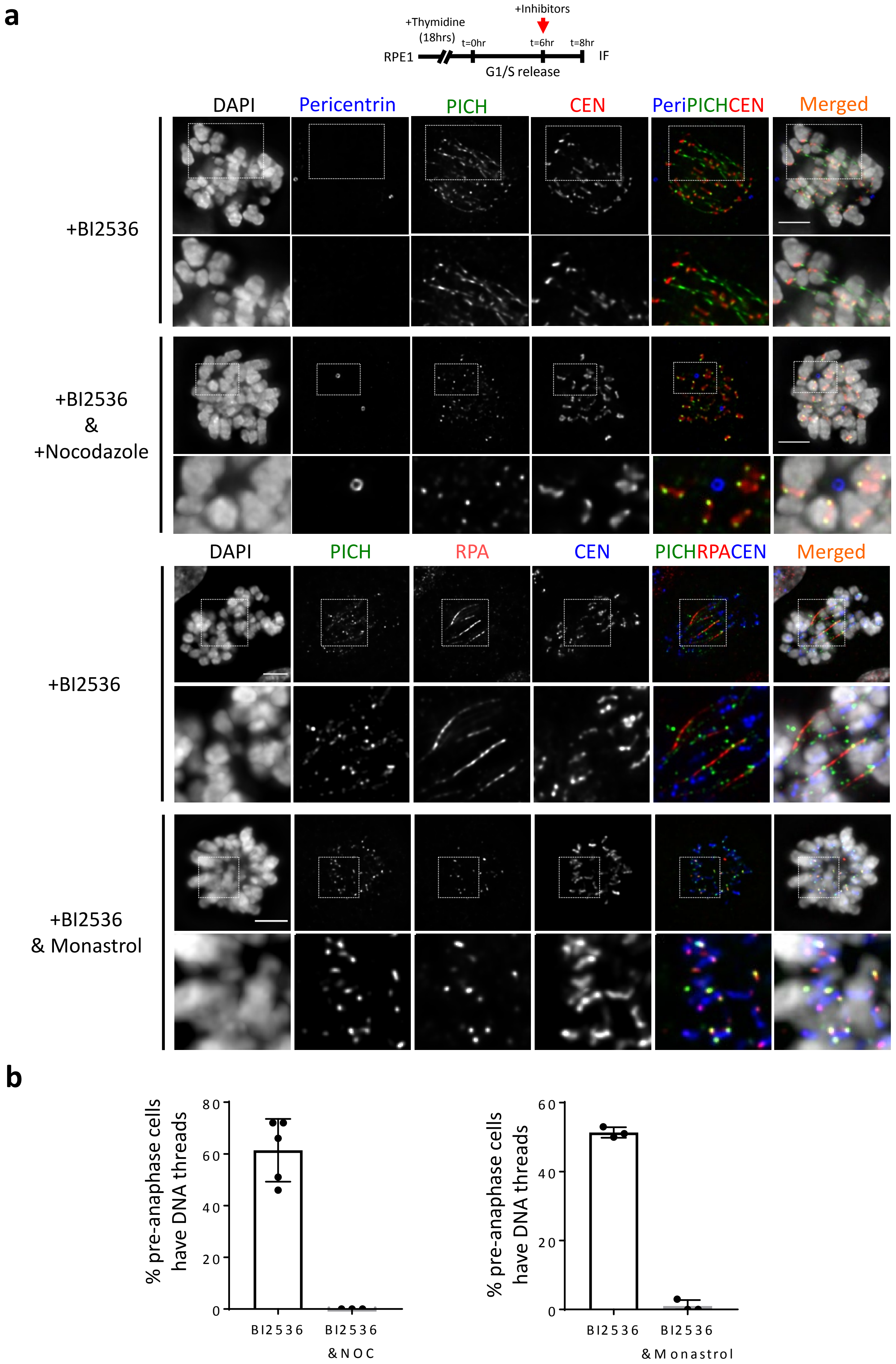

Supplementary Figure 8. Addis Jones *et al.*

Supplementary Figure 8. Bipolar spindle-tension is required for DNA thread formation induced by PLK1 inactivation

(a) Experimental outline and representative images showing the abolishment of bipolarity, or spindle-tension prevent the DNA thread formation induced by PLK1 inactivation. Co-treatment of either nocodazole (a spindle poison) or monastrol (Eg5 inhibitor) abolishes DNA threads formation. Scale bar=5μm. **(b)** Quantification of DNA thread formation after the indicated treatment (n=3 independent experiments analysing a total of 601 and 382 cells in BI2536 and BI2536+nocodazole conditions, respectively; n=3 independent experiments analysing a total of 336 and 274 cells in BI2536 and BI2536+monastrol conditions, respectively; mean ± S.D. is shown).

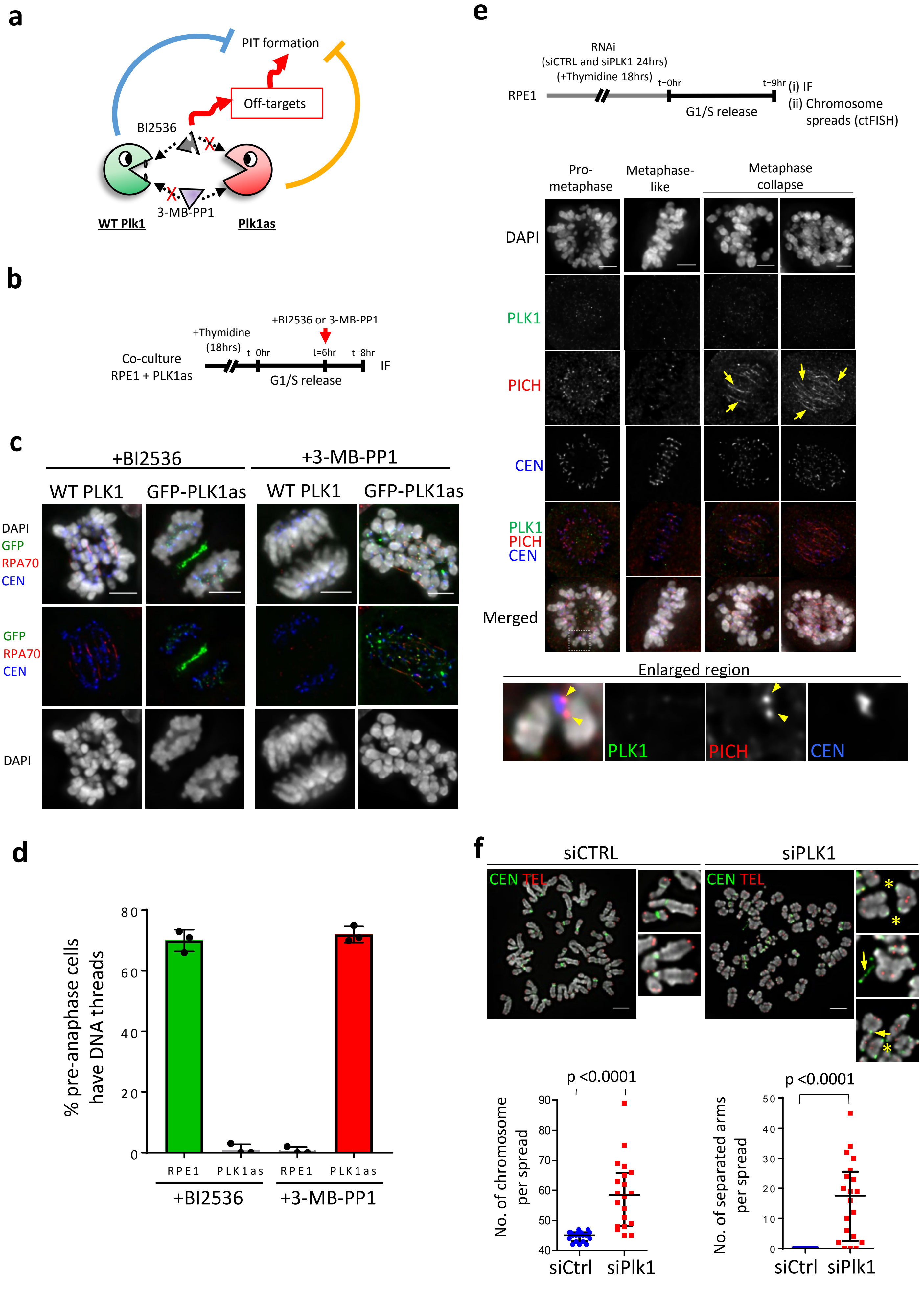

Supplementary Figure 9. Addis Jones *et al.*

Supplementary Figure 9. PLK1 kinase activity is required to suppress centromeric DNA thread formation and 'centromere dislocations'.

a) A diagram depicting the potential off-target effects of BI2536 on centromere DNA thread formation (e.g. PLK2, PLK3 & PLK4 inhibition), and at the same time, demonstrating how PLK1as cells are insensitive to BI2536, whilst providing sensitivity to PLK1 activity via 3-MB-PP1 addition. **(b)** Experimental outline of both wildtype RPE1 and GFP-tagged RPE1 PLK1as cells for examining DNA thread formation following the treatment of either BI2536 [60nM] or 3-MB-PP1 analogue [1μM]. **(c)** Representative images display both wildtype RPE1 and GFP-tagged PLK1as RPE1 mitotic cells, showing DNA threads following BI2536 (WT), or 3-MB-PP1 (PLK1as) treatment. **(d)** Quantification of DNA thread formation after BI2536 or 3-MB-PP1 treatment in parental and GFP-PLK1as RPE1 cells (n=3 independent experiments analysing a total of 224, 168, 148 and 300 cells in the indicated conditions; mean ± S.D. is shown). **(e)** Experimental outline for both cell synchronisation steps and combined siRNA oligo targeting of PLK1, before immunofluorescent and chromosome spread preparation. Representative images of PLK1-depleted RPE1 cells forming PICH-coated DNA threads (arrows). Enlarged region (below; arrowheads) denotes the presence of PICH protein localisation at the centromere, despite the absence of PLK1. **(f)** ctFISH analysis of chromosome spreads from control (siCTRL) and PLK1-depleted (siPLK1) RPE1 cells. Quantifications show the numbers of chromatin (left), and 'centromere dislocations' (right) (19 and 20 mitotic spreads of siCtrl and siPlk1 treated RPE1 cells were analysed respectively; mean± S.E.M. is shown; scale bars=5μm).

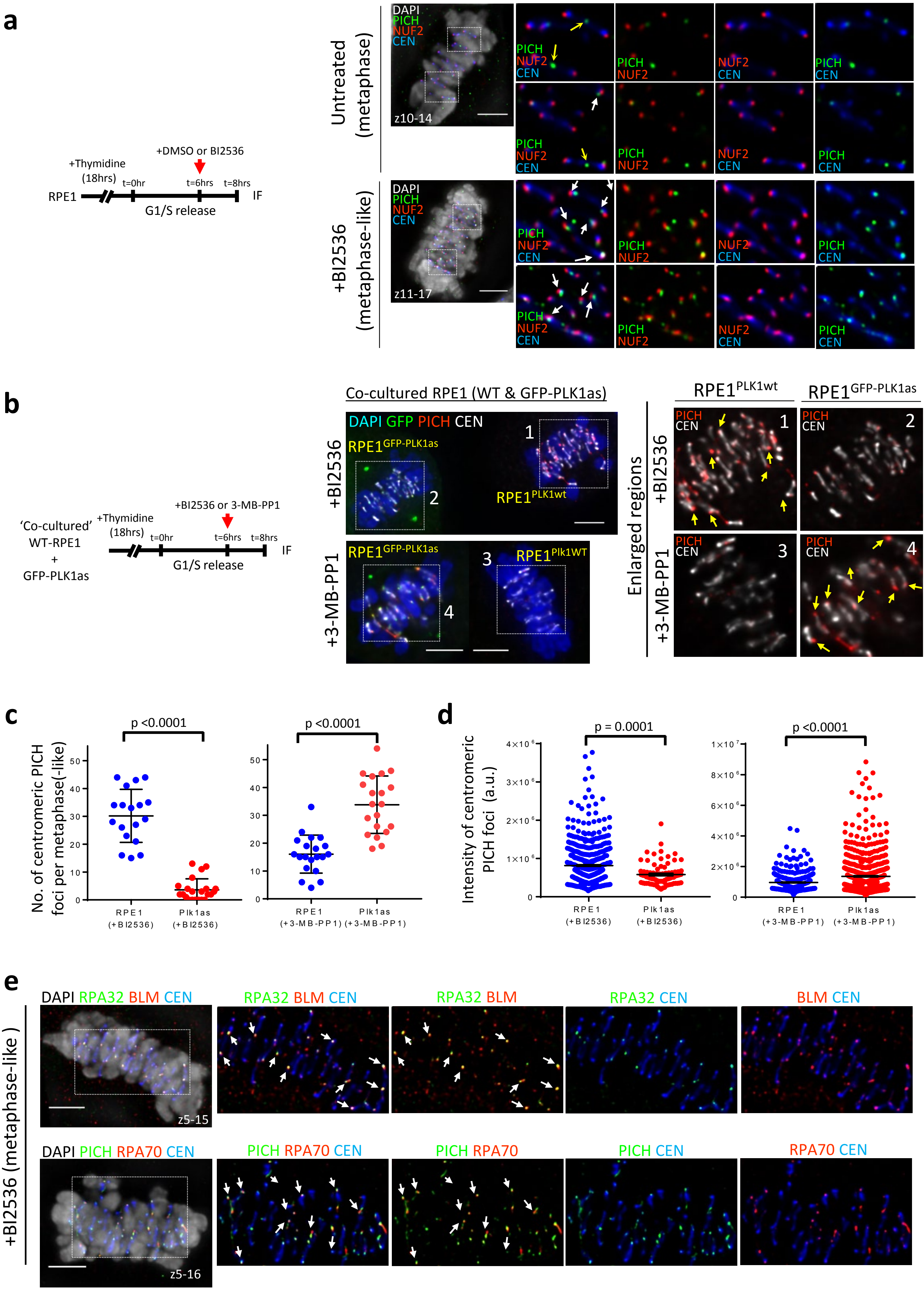

Supplementary Figure 10. Addis Jones *et al.*

Supplementary Figure 10. Loss of PLK1 induces aberrant accumulation of PICH, BLM and RPA at kinetochores before metaphase collapse.

(a) Experimental outline of cell synchronisation and drug treatment (left). Representative images of metaphase (untreated) and metaphase-like (BI2536) cells stained with PICH, NUF2 and centromeres. White arrows denote aberrant PICH accumulation at centromeres after BI2536 treatment. Note: PICH was also occasionally observed at the inner-centromeres of untreated cells (yellow arrows) **(b)** Experimental outline of cell synchronisation and drug treatment on co-cultured wild-type RPE1 cells and RPE1 GFP-PLK1as cells. Representative images showing the induction of centromeric PICH foci after PLK1 inhibition in wild-type RPE1 and GFP-PLK1as cells following the corresponding BI2536 or 3-MB-PP1 treatment. Right panels: enlarged regions showing strong PICH foci in the corresponding treatment. Quantification of PICH foci numbers **(c)** (18, 22, 20 and 20 cells in the indicated conditions were analysed; mean $\pm$ S.D. is shown) and intensities **(d)** (543, 79, 321 and 676 centromeric PICH foci were measured in the indicated conditions; mean $\pm$ S.E.M. is shown). Metaphase(-like) cells were analysed from co-cultures of wild-type RPE1 and PLK1as cells, after treatment with either BI2536 (left) or 3-MB-PP1 (right) . **(e)** Representative images showing co-localisation of PICH, BLM and RPA foci at centromeres induced by BI2536 in metaphase(-like) RPE1 cells. Scale bars=5μm.

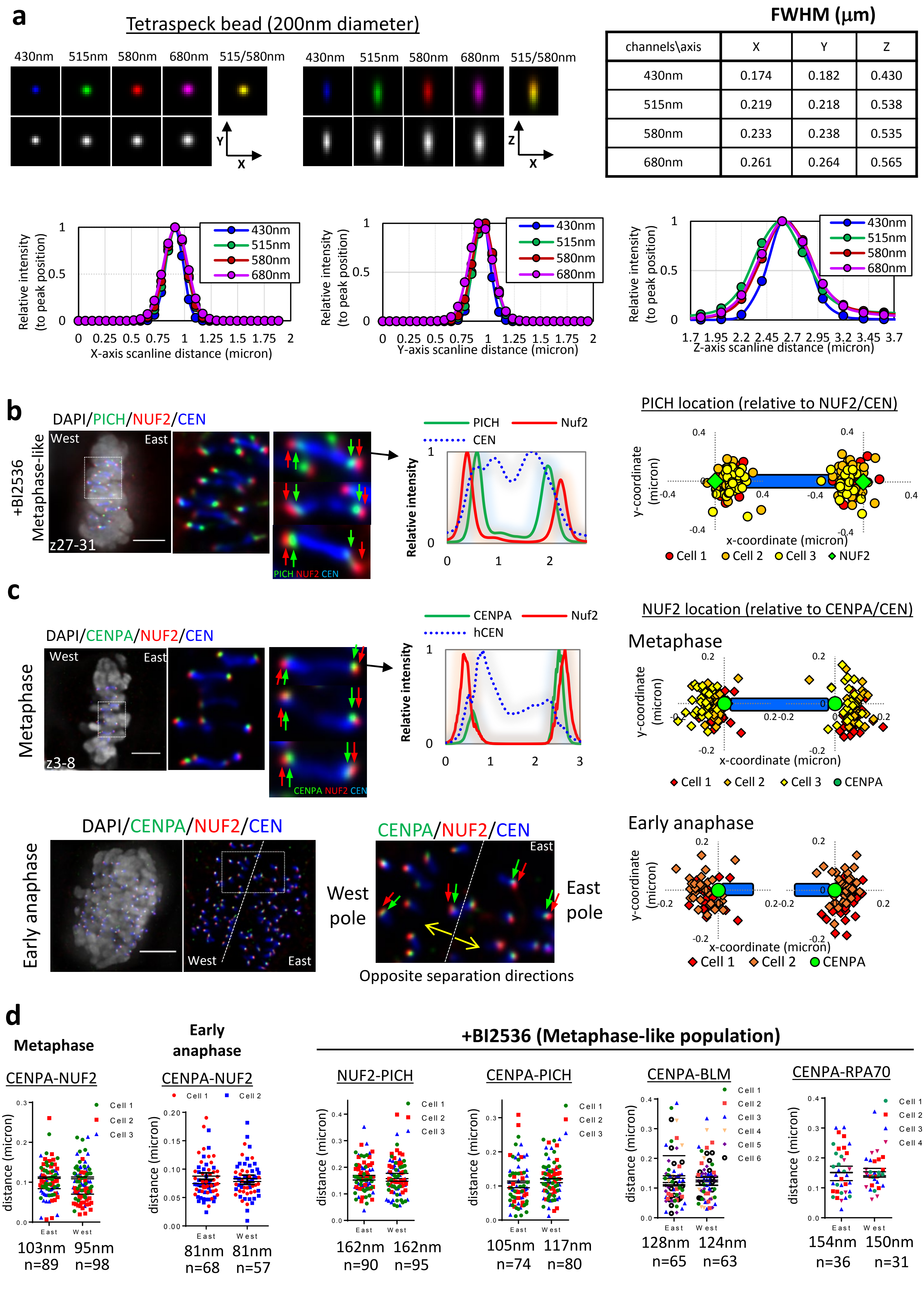

channels\axis	X	Y	Z
430nm	0.174	0.182	0.430
515nm	0.219	0.218	0.538
580nm	0.233	0.238	0.535
680nm	0.261	0.264	0.565

Supplementary Figure 11. Addis Jones *et al.*

Supplementary Figure 11. High-resolution and -precision deconvolution microscopy reveals UFB-binding complex underneath kinetochores.

(a) Images of a 4-color TetraSpeck bead (left) of 200nm diameter in X, Y and Z. Table showing the full width at half maximum (FWHM) (right) of deconvolved TetraSpeck bead images. Scanline plots (below) show the alignments of X, Y and Z positions of a TetraSpeck bead. **(b)** Representative image of a metaphase(-like) cell treated with BI2536. Image, plot-profile and graph (left to right) demonstrate the relative locations of PICH, compare to NUF2, in East and West poles of the centromere. **(c)** Representative images of the locations of NUF2 relative to CENPA in metaphase and early anaphase cells. **(d)** Relative distances of CENPA to NUF2, in East and West poles of centromeres from untreated RPE1 metaphase and early anaphase cells (left two graphs). Relative distances of NUF2 to PICH, CENPA to PICH, CENPA to BLM and CENPA to RPA in East and West poles of the centromere from metaphase(-like) cells after BI2536 treatment (right four graphs) (n shows the numbers of foci analysed from the indicated number of cells; mean distance of each analysed cell is shown). Scale bars=5μm.

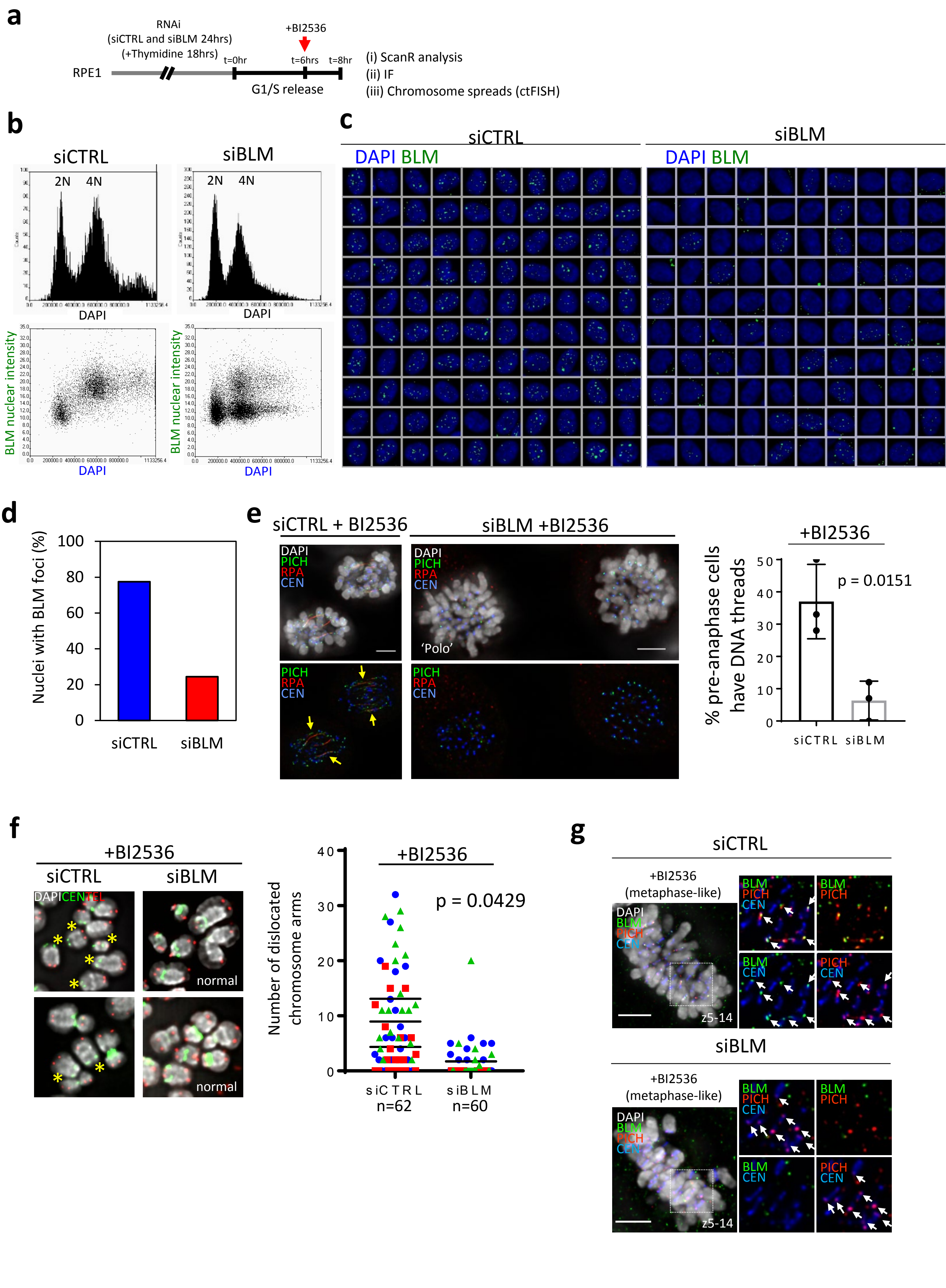

Supplementary Figure 12. Addis Jones *et al.*

Supplementary Figure 12. BLM depletion reduces both centromere DNA thread formation and 'centromere dislocations' induced by PLK1 inactivation.

(a) Experimental outline of BLM RNAi, cell synchronisation and treatment in RPE1 cells. **(b)** Cell-cycle profile plot (top) of DAPI intensity and scatter plot (below) of DAPI intensity against BLM nuclear intensity using ScanR high-content screening microscopy on control (siCTRL) and BLM-depleted (siBLM) samples. Note: BLM expression is cell-cycle regulated and siBLM generates a G2/M population with low BLM nuclear intensity. **(c)** Representative image gallery of 100 random cell nuclei as from (b), showing BLM nuclear foci in siCTRL and siBLM-treated RPE1 cells. **(d)** Graph to show the overall percentage of nuclei positive for BLM foci, as from (b) (7462 and 12637 cells from siCTRL and siBLM-treated cells were measured, respectively). **(e)** Representative images and quantification of pre-anaphase RPE1 cells forming DNA threads in siCTRL and siBLM oligos treatment after BI2536 treatment (n=3 independent experiments analysing 304 and 326 cells in siCTRL and siBLM conditions respectively; mean $\pm$ S.D. is shown). **(f)** Representative chromosome images and quantification of 'centromere dislocations' in siCTRL and siBLM oligos treatment after BI2536 treatment (n=3 independent experiments analysing 62 and 60 mitotic spreads in siCTRL and siBLM conditions respectively; mean of each experiment is shown). **(g)** Representative images showing the reduction of centromeric BLM, but not PICH, foci in metaphase(-like) cells after RNAi treatment as done in (a). Scale bar=5μm.

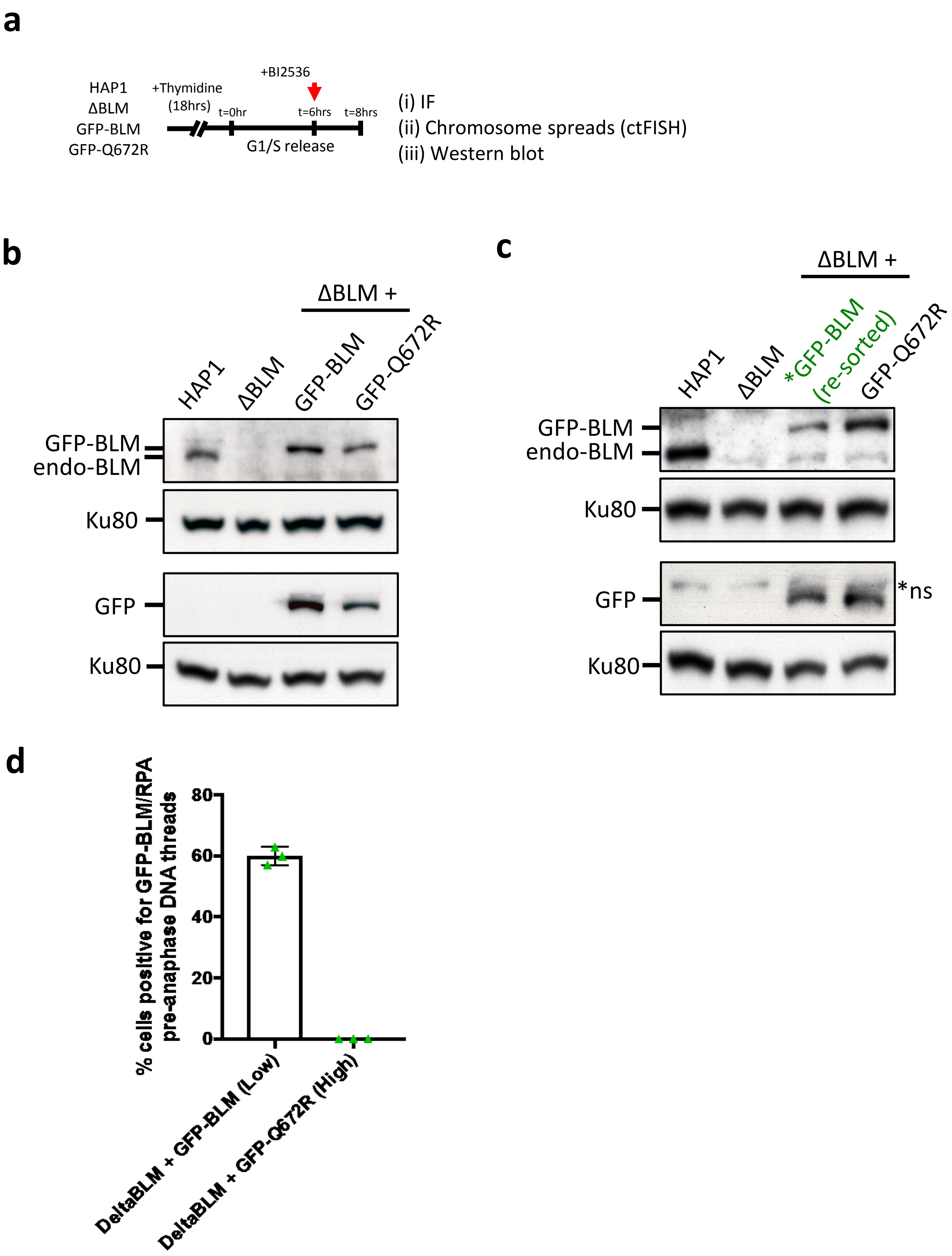

Supplementary Figure 13. Addis Jones *et al.*

Supplementary Figure 13. BLM-helicase activity is responsible for centromeric DNA unwinding during PLK1 inactivation.

(a) Experimental outline for cell synchronisation and BI2536 treatment, in HAP1 cells. **(b)** Western blot showing BLM protein levels in HAP1, ΔBLM HAP1 cells, and stable cell lines derived from ΔBLM cells, expressing either a wild-type GFP-BLM, or a helicase mutant GFP-Q672R. Antibodies were used to target either BLM (top), or GFP (bottom). Anti-KU80 was used as a loading control. **(c)** Western blot showing BLM protein levels in the HAP1 stable cell lines expressing GFP-BLM and GFP-Q672R after re-sorting by FACS. Note: the wild-type GFP-tagged BLM (GFP-BLM) expression is lower than the GFP-Q672R mutant. Antibodies were used to target either BLM (top) or GFP (bottom). Anti-KU80 was used for loading control (*ns=non-specific antibody detection). **(d)** Quantification of centromere DNA thread formation in the FACS re-sorted cells from (c). Note: a low expression of wildtype GFP-BLM ('low expression') but not GFP-Q672R ('high expression') induced DNA thread formation after BI2536 treatment (n=3 independent experiments analysing a total of 303 and 291 cells of GFP-BLM and GFP-Q672R cell lines, respectively; mean ± S.D. is shown).

a

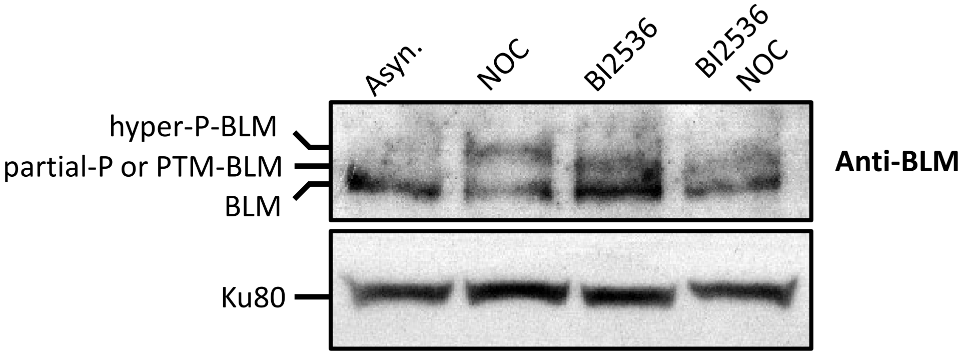

b

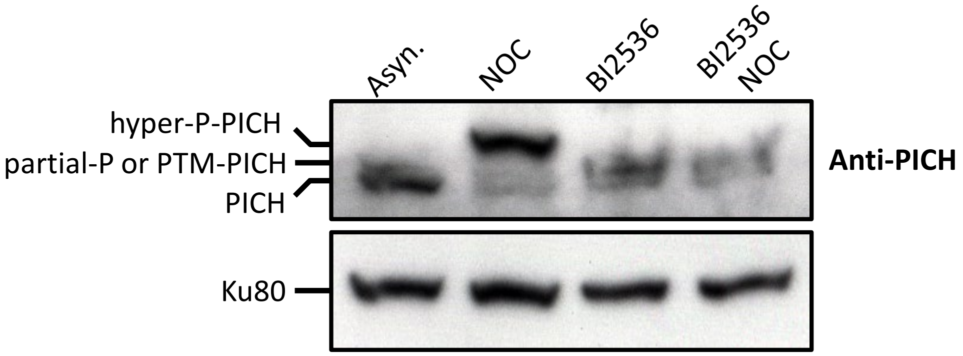

Supplementary Figure 14. Mitotic hyperphosphorylation of BLM and PICH is partially dependent on PLK1 activity.

(a) Western blot image showing different migration forms of BLM protein in RPE1 cells after the indicated treatment for 16 hours. **(b)** same treatment as in (a), but probed with anti-PICH antibody. Ku80 was used as a loading control.

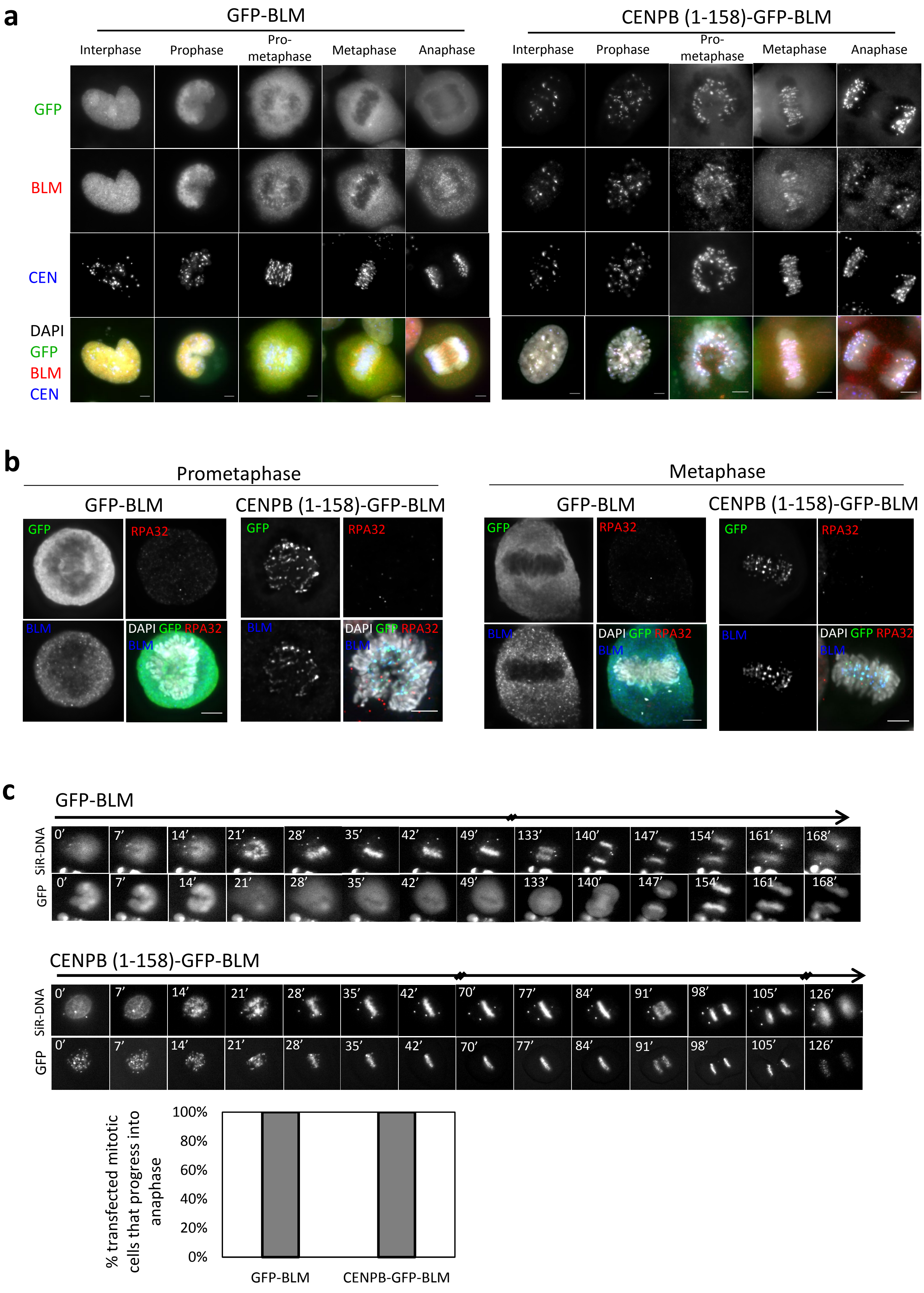

Supplementary Figure 15. Addis Jones *et al.*

Supplementary Figure 15. Artificial tethering of BLM at the centromere in HeLa cells does not trigger metaphase collapse.

(a) HeLa cells were transiently transfected with a wildtype GFP-BLM or a CENPB(1-158) fused GFP-BLM protein (CENPB-GFP-BLM). Representative images of HeLa cells at various stages of mitosis showing the localisation of GFP-BLM (left panels) and CENPB-GFP-BLM protein (right panels). **(b)** Representative images of RPA staining in the transfected HeLa mitotic cells described in (a). **(c)** Time-lapse microscopy images (top) and quantification (bottom) of the transfected HeLa cells progressing in mitosis. DNA was stained by Sir-DNA (15 and 56 transfected prophase cells of GFP-BLM and CENPB-GFP-BLM cell lines were analysed respectively; Scale bars=5μm).
