# Supplementary material for: PLK1 facilitates chromosome biorientation by suppressing centromere disintegration driven by BLM-mediated unwinding and spindle pulling
Source: Nat Commun. 2019 Jun 28;10:2861. doi: 10.1038/s41467-019-10938-y (PMC6599003; doi:10.1038/s41467-019-10938-y)
Supplement: Supplementary file 9 — Reporting Summary [file 41467_2019_10938_MOESM9_ESM.pdf]

nature research

Corresponding author(s): Kok-Lung Chan

Last updated by author(s): May 29, 2019

Reporting Summary

Nature Research wishes to improve the reproducibility of the work that we publish. This form provides structure for consistency and transparency in reporting. For further information on Nature Research policies, see Authors & Referees and the Editorial Policy Checklist.

Statistics

For all statistical analyses, confirm that the following items are present in the figure legend, table legend, main text, or Methods section.

n/a	Confirmed	
☐	☒	The exact sample size (*n*) for each experimental group/condition, given as a discrete number and unit of measurement
☒	☐	A statement on whether measurements were taken from distinct samples or whether the same sample was measured repeatedly
☐	☒	The statistical test(s) used AND whether they are one- or two-sided<br>*Only common tests should be described solely by name; describe more complex techniques in the Methods section.*
☒	☐	A description of all covariates tested
☒	☐	A description of any assumptions or corrections, such as tests of normality and adjustment for multiple comparisons
☐	☒	A full description of the statistical parameters including central tendency (e.g. means) or other basic estimates (e.g. regression coefficient) AND variation (e.g. standard deviation) or associated estimates of uncertainty (e.g. confidence intervals)
☒	☐	For null hypothesis testing, the test statistic (e.g. *F*, *t*, *r*) with confidence intervals, effect sizes, degrees of freedom and *P* value noted<br>*Give P values as exact values whenever suitable.*
☒	☐	For Bayesian analysis, information on the choice of priors and Markov chain Monte Carlo settings
☒	☐	For hierarchical and complex designs, identification of the appropriate level for tests and full reporting of outcomes
☒	☐	Estimates of effect sizes (e.g. Cohen's *d*, Pearson's *r*), indicating how they were calculated

Our web collection on statistics for biologists contains articles on many of the points above.

Software and code

Policy information about availability of computer code

Data collection	No such software used.
Data analysis	ImageJ measurement of kinetochore foci coordinates, distances and intensities<br>Spot Pair Distance Tool<br>Measures the distance between spots in 2 channels of an image. The tool searches within a focus/box radius, typically +/-5px, for a local maxima in the two pre-selected analysis channels. The centre-of-mass around each maxima, typically +/- 2px, is computed as the centre of intensity for each channel. Dragging from the clicked point creates a reference direction. The Euclidean distance between the centres is reported, optionally with the signed XY distance and angle relative to the reference direction. Visual guides are overlaid on the image to assist in spot selection and direction orientation. Available in the latest GDSC ImageJ plugins.<br><br>Spot Fit Tool<br>Fits a 2D Gaussian to a spot in an image. The tool searches within a box radius, typically +/-3px, for a local maxima in the pre-selected analysis channel. A 3x3 smoothing filter is applied before identification of the maxima. A 2D Gaussian function is then fitted to the data using non-linear least-squares fitting and poor fits rejected using a signal-to-noise ratio. The 18 parameters for the fit are reported including the total intensity under the Gaussian function and the local background value. Visual guides are overlaid on the image to show the fitted location. Available in the pre-release GDSC SMLM ImageJ plugins.

For manuscripts utilizing custom algorithms or software that are central to the research but not yet described in published literature, software must be made available to editors/reviewers. We strongly encourage code deposition in a community repository (e.g. GitHub). See the Nature Research guidelines for submitting code & software for further information.

Data

Policy information about availability of data

All manuscripts must include a data availability statement. This statement should provide the following information, where applicable:

- Accession codes, unique identifiers, or web links for publicly available datasets
- A list of figures that have associated raw data
- A description of any restrictions on data availability

The authors declare that the data supporting the findings of this study are available within the paper and its supplementary information files. Raw imaging data are available from the corresponding author upon reasonable request.

Field-specific reporting

Please select the one below that is the best fit for your research. If you are not sure, read the appropriate sections before making your selection.

☒ Life sciences ☐ Behavioural & social sciences ☐ Ecological, evolutionary & environmental sciences

For a reference copy of the document with all sections, see nature.com/documents/nr-reporting-summary-flat.pdf

Life sciences study design

All studies must disclose on these points even when the disclosure is negative.

Sample size	Sample size was comparative to previous cell biology based studies of individual cell analyses, whilst also giving confidence of a fair statistical outcome.
Data exclusions	No data was excluded.
Replication	All relevant experiments were completed a minimum of three times, unless stated otherwise. All repeats were also successful.
Randomization	Experiments were not randomized.
Blinding	No specific blinding was taken during experiments and analyses. However, in general, repeats were completed independently without influence from other authors.

Reporting for specific materials, systems and methods

We require information from authors about some types of materials, experimental systems and methods used in many studies. Here, indicate whether each material, system or method listed is relevant to your study. If you are not sure if a list item applies to your research, read the appropriate section before selecting a response.

Materials & experimental systems

n/a	Involved in the study	
☐	☒	Antibodies
☐	☒	Eukaryotic cell lines
☒	☐	Palaeontology
☒	☐	Animals and other organisms
☒	☐	Human research participants
☒	☐	Clinical data

Methods

n/a	Involved in the study	
☒	☐	ChIP-seq
☐	☒	Flow cytometry
☒	☐	MRI-based neuroimaging

Antibodies

Antibodies used

Primary antibodies:
anti-PICH (Abnova; H00054821-B01P)
anti-PICH (Abnova; H00054821-D01P)
anti-BLM (Santa Cruz; sc-7790)
anti-BLM (Abcam; ab2179)
anti-gH2AX (Upstate; JBW-301)
anti-TOP2A (Santa Cruz; sc-5348)
anti-SMC2 (Bethyl Lab; A300-058A)
anti-RPA70 (Abcam; ab79398)
anti-RPA32 (Abcam; ab2175)
anti-CENPA (Abcam; ab13939)
anti-CENPB (Abcam; ab25734)

anti-NUF2 (Abcam; ab122962)
anti-PLK1 (Santa Cruz; sc-55504)
anti-pericentrin (Abcam; ab4448)
anti-centromere (ImmunoVision; HCT-0100)
GFP booster (ChromoTek; gba-488)

Secondary antibodies:
donkey anti-mouse Alexa Fluor 488, 555 and 647
donkey anti-rabbit Alexa Fluor 488, 555 and 647
donkey anti-goat Alexa Fluor 488 and 555
goat anti-human DyLight 550 and 650
All secondary antibodies were purchased from ThermoFisherScientific

Validation	Validation data is provided by each manufacturer online.

Eukaryotic cell lines

Policy information about cell lines

Cell line source(s)	RPE1-hTERT, 82-6-hTERT, 1BR3 primary fibroblasts, HeLa and HCT116 colon cancer cells were obtained from the Genome Damage and Stability Centre Cell Bank. RPE1-hTERT and HCT116 cells were originally purchased from ATCC. RPE1-hTERT derivative cells (RPE1-GFP-PLK1/PLK1as) were generated and supplied by Mark Burkard (University of Wisconsin). Bloom’s syndrome fibroblasts (GM08505) were obtained from Phillip North (University of Oxford). HAP1 cells and HAP1 ΔBLM cells were obtained from Marcel van Vugt (University of Groningen).
Authentication	All cell lines were authenticated by STR genotyping from European Collection of Cell Cultures
Mycoplasma contamination	All cell lines were tested and were negative for Mycoplasma contamination (Lonza Mycoplasma testing kit)
Commonly misidentified lines (See ICLAC register)	No ICLAC registered misidentified cell lines were used.

Flow Cytometry

Plots

Confirm that:

☐ The axis labels state the marker and fluorochrome used (e.g. CD4-FITC).

☒ The axis scales are clearly visible. Include numbers along axes only for bottom left plot of group (a 'group' is an analysis of identical markers).

☐ All plots are contour plots with outliers or pseudocolor plots.

☐ A numerical value for number of cells or percentage (with statistics) is provided.

Methodology

Sample preparation	Cells were trypsinised, washed with PBS and fixed with 70% ice-cold ethanol. For cell cycle analysis, cells were washed with PBS and re-suspended in propidium iodide (PI)/RNaseA staining buffer.
Instrument	BD Acurri C6
Software	BD C Sampler
Cell population abundance	*Describe the abundance of the relevant cell populations within post-sort fractions, providing details on the purity of the samples and how it was determined.*
Gating strategy	*Describe the gating strategy used for all relevant experiments, specifying the preliminary FSC/SSC gates of the starting cell population, indicating where boundaries between "positive" and "negative" staining cell populations are defined.*

☐ Tick this box to confirm that a figure exemplifying the gating strategy is provided in the Supplementary Information.
